# Evaluation of a clinically intuitive quality assurance method

**DOI:** 10.1088/1742-6596/444/1/012022

**Published:** 2013

**Authors:** H Norris, A Thomas, M Oldham

**Affiliations:** Duke University Medical Center, Durham, USA

## Abstract

There is a pressing need for clinically intuitive quality assurance methods that report metrics of relevance to the likely impact on tumor control of normal tissue injury. This paper presents a preliminary investigation into the accuracy of a novel “transform method” which enables a clinically relevant analysis through dose-volume-histograms (DVHs) and dose overlays on the patient’s CT data. The transform method was tested by inducing a series of known mechanical and delivery errors onto simulated 3D dosimetry measurements of six different head-and-neck IMRT treatment plans. Accuracy was then examined through the comparison of the transformed patient dose distributions and the known actual patient dose distributions through dose-volume histograms and normalized dose difference analysis. Through these metrics, the transform method was found to be highly accurate in predicting measured patient dose distributions for these types of errors.

## 1. Introduction

With the advent of new, more complex treatment methods in radiation therapy, the need for a 3D quality assurance method has become apparent [1, 2]. New devices have been introduced which make a “semi-3D” measurement with diode arrays; extensive studies have been performed evaluating the accuracy of these devices [3]. Oldham et al [4] recently reported a novel approach to clinically intuitive QA which yields clinical metrics from 3D dose measurements made in phantom. The method was retrospectively applied to 6 patients that received base-of-skull (BOS) IMRT treatment. Although good agreement was observed between the measured and planned DVHs in these cases, the uncertainty in the measured DVH curves was unknown. The present work characterizes and investigates this uncertainty intentionally introducing known errors to the same set of patients.

## 2. Methods

**Figure 1:**
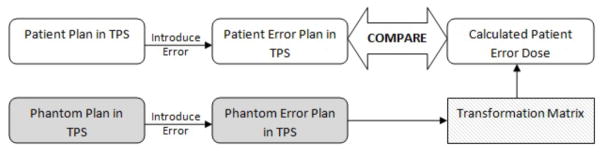
Schematic of methods; error-induced phantom dose distribution is used to calculate the transformed patient dose distribution, which is compared to the error-induced patient dose distribution. TPS refers to treatment-planning-system.

An assortment of mechanical, calibration and delivery errors were introduced into the treatment plan of each of the six patients. These included ±5° errors on the gantry, couch, and collimator for all beams, and 105% or 95% of the prescription dose. A total of 8 error-induced plans were therefore created for each patient. The *patient error dose distributions* corresponding to these errors were calculated using Eclipse Analytic Anisotropy Algorithm (AAA) [5] at 2.5 mm^3^ isotropic resolution, with the heterogeneity correction applied. These dose distributions serve as the actual dose that would be delivered to the patient should these errors actually occur during delivery; they are the “gold standard” to which the transformed dose distributions will later be compared. Corresponding *phantom verification dose distributions* were calculated on an RPC-like anthropomorphic head phantom. These dose distributions act as the simulated 3D dosimetry measurements. The *transformed dose distribution* in the patient geometry is calculated with the transformation matrix, as described in Oldham et al. [4]. A schematic outline of the simulation rationale is shown in Figure 1.

The accuracy of the transform method was evaluated by comparing the transformed dose distribution (upper right box in figure 1) with the gold standard, the patient error dose distribution. Clinically relevant metrics such as patient dose-volume histograms were obtained as well as 3D gamma passing rates and the Normalized Dose Distribution (NDD – a variation of gamma) [6].

## 3. Results

### 3.1. Normalized Dose Difference Based Metrics

The NDD values were calculated over both the entire patient volume for a global passing rate as well as for the PTV and a series of critical structures: the brainstem, the optic chiasm, the left and right eyes, and the left and right optic nerves. 2% dose difference and 2 mm distance to agreement were used. For the NDD metric, voxel values between −1 and +1 are passing.

**Figure 2:**
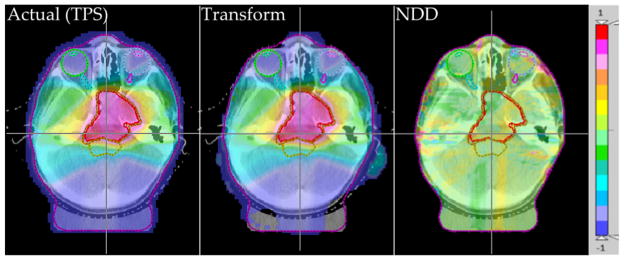
Actual (TPS) (left) and transformed (center) relative dose distributions with the 2%/2mm NDD analysis of the two (right), for patient HN4 with Gantry +5°.

Figure 2 shows an example of a trans-axial slice of patient case HN4 with induced error gantry +5° for all beams, overlaid with the actual dose distribution, the transformed dose distribution, and the NDD analysis of the two. The passing rate NDD values are 100% for all structures and 99.08% globally.

Overall, NDD metrics comparing the transformed dose distribution with the actual dose showed very good agreement with the actual dose distributions when the NDD was performed with 2% dose difference, 2 mm distance-to-agreement criteria. The delivery errors where the dose normalization was changed by ±5% of the prescription dose had 100% pass rates for the global NDD as well as for each structure. The mechanical errors also showed very good passing rates for the same 2%/2mm criteria, especially for the collimator and couch errors, where the global passing rates were very high and the passing rates to the structures were at or near 100% for all patient plans. The gantry errors had the lowest NDD passing rates for the 2%/2mm criteria, but in general these passing rates were still very high (>98%).

### 3.2. Dose-Volume Histogram Based Metrics

**Figure 3:**
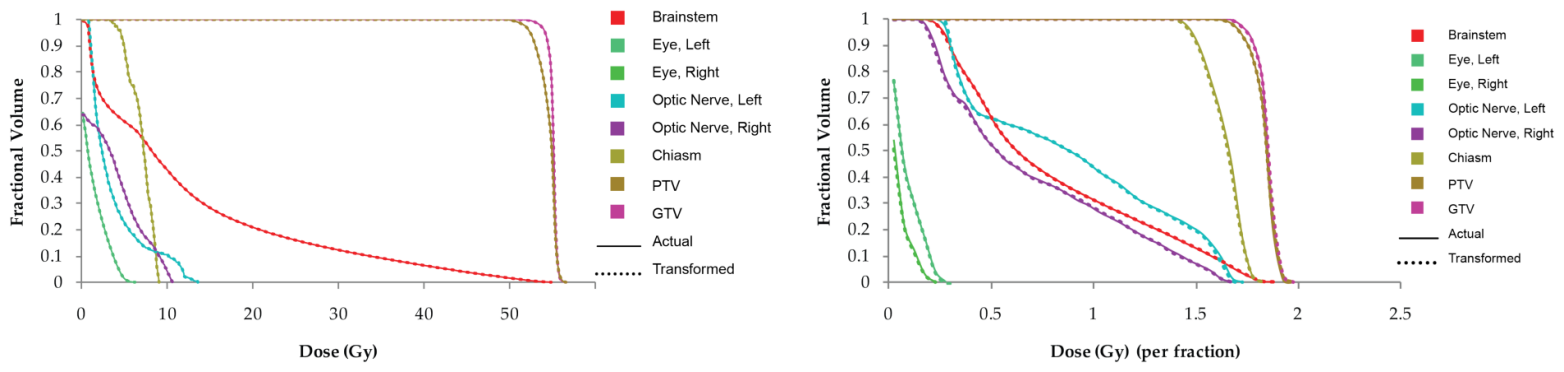
Best DVH agreement is seen for the dose delivery errors, as seen in patient HN1 with the prescription dose +5% (left); worst DVH agreement is seen for patient HN4 with the gantry+5°.

With the overlaid transformed dose distribution on the patient CT set, a DVH can be calculated and compared to the actual DVH from the patient error dose distribution. This was done for all cases; the results were similar over all induced mechanical errors. Figure 3 shows an example of this for the prescription dose +5% on patient HN1 and the gantry rotated +5° on patient case HN4.

In general, the DVHs showed very good agreement between the transformed and the actual dose distributions. The delivery errors, where the plan normalization was increased or decreased by 5% of the planned prescription dose, showed indistinguishable DVH lines, indicating that the transform method produced perfect or near-perfect dose distributions. The mechanical errors, where the collimator, the couch, or the gantry were rotated ±5° from the planned values, also showed very good agreement in the DVH lines, although some slight deviations could be seen.

**Figure 4:**
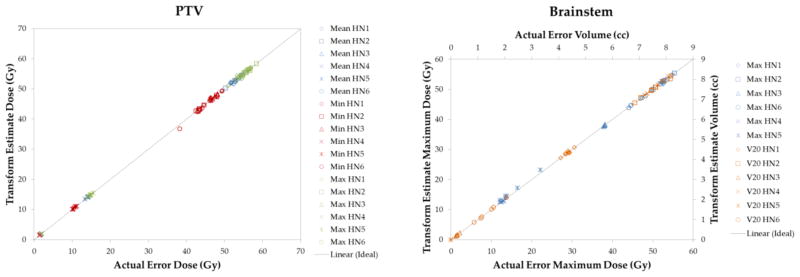
The maximum, minimum, and mean dose delivered to the PTV for all cases; transformed dose vs. error-induced TPS dose (left); maximum dose and V_20_ for the brainstem; transformed estimate dose vs. actual dose (right).

From the DVHs for all the plans, the minimum, maximum, and mean doses to the PTV were found for both the error-induced plan from the TPS and the transformed dose distribution. Correlations between the transformed dose values and error-induced planned dose values can be found by plotting the transformed dose value versus the error-induced planned dose value. Figure 4 shows this plot for the PTV as well as for the brainstem. These data show a high level of agreement between the transformed and the “gold standard” dose distributions.

For the PTV, the maximum dose has a linear fit given by y = 1.0004x, with R^2^ = 0.9998; for the minimum dose, y = 0.9968x and R^2^ = 0.9995; for the mean dose, the line of best fit is given by y = 0.9997x and R^2^ = 0.9999. The line of best fit for the maximum dose to the brainstem is given by y = 0.9987x with R^2^ = 0.9999. The line of best fit for the V_20_ data is given by y = 0.9999x with R^2^ = 0.9999. These values again reflect a high level of accuracy between the actual and the transformed dose distributions.

## 4. Discussion

A high degree of accuracy was seen in the transform method, even when presented with relatively large mechanical and delivery errors. This is evidenced in the agreement of dose-volume histogram lines between the transformed and the actual dose distributions, as well as very high NDD passing rates under stringent criteria of 2% dose difference and 2 mm distance to agreement. This high level of accuracy in the transform method leads to the feasibility of performing clinically-relevant analysis of patient-specific quality assurance.

## 5. Conclusions

The transform method was shown to work well for the types of errors that were induced - rotating the gantry, couch, and collimator each ±5° and changing the prescription dose ±5%. These errors were rather large, certainly beyond what would be typically seen within the clinic, but the transform method continued to perform with a high level of accuracy. This level of accuracy can be attributed in part to the high resolution of 3D dosimetry.

The transform method is limited in clinical use by the size of the dosimeter. This can affect the transformed values for the organs at risk; if a PTV is large or if its volume is not completely contained within the dosimeter, then the accuracy of the transform method will be greatly reduced. In addition, this study is limited to head-and-neck/base-of-skull IMRT treatments. Further investigation focus on the accuracy of the method for a wider range of scenarios, and other sites with greater sources of inhomogeneity.
